# Characterization analyses of *MADS-box* genes highlighting their functions with seed development in *Ricinus communis*

**DOI:** 10.3389/fpls.2025.1589915

**Published:** 2025-05-14

**Authors:** Jing Sun, Zekun Zhou, Fanqing Meng, Mengyun Wen, Aizhong Liu, Anmin Yu

**Affiliations:** ^1^ Key Laboratory for Forest Resources Conservation and Utilization in the Southwest Mountains of China, Ministry of Education, Southwest Forestry University, Kunming, China; ^2^ Yunnan Provincial Key Laboratory for Conservation and Utilization of In-forest Resource, Southwest Forestry University, Kunming, China; ^3^ Yunnan Key Laboratory of Crop Wild Relatives Omics, Kunming Institute of Botany, Chinese Academy of Sciences, Kunming, China

**Keywords:** MADS-box, co-expression, seed development, regulators, castor

## Abstract

The *MADS-box* gene family plays a pivotal role in regulating floral organ development and various aspects of plant growth. Despite its well-established importance in many species, the function and evolution of *MADS-box* genes in *Ricinus communis* (castor) remain unexplored. This study presents an extensive genome-wide analysis of the *MADS-box* gene family in castor, covering their physicochemical characteristics, phylogenetics, gene architecture, chromosomal distribution, evolutionary dynamics, expression profiles, and co-expression networks. In total, 56 *MADS-box* genes were categorized into two main phylogenetic groups: type-I and type-II, which were further subdivided into three and two subgroups, respectively. Segmental duplication was found to be the primary driver of *MADS-box* gene expansion in castor, while purifying selection was evident across the entire gene family, as indicated by the *Ka*/*Ks* ratio. In-depth analyses of gene expression, promoter motifs, co-expression networks, and experimental validation (Y1H assays and qRT-PCR) revealed that *RcMADS16* and *RcMADS4*1 are key regulators of castor seed development, with *RcMADS16* may involve in seed coat formation and *RcMADS41* in oil accumulation. This study not only provides the first detailed insight into the evolutionary and functional landscape of *MADS-box* genes in castor, but also establishes a foundation for future investigations into the role of these genes in seed and organ development, both in castor and other plant species.

## Introduction

*Ricinus communis* L. (castor) is one of the most important non-edible oil crops in worldwide, contributing significantly to biodiesel feedstock through its high hydroxy fatty acid content. Originating in Africa or India, castor bean was domesticated approximately 3,200 years ago in East Africa (Xu et al., 2021). Nowadays, castor bean is primarily cultivated in regions, such as South Asia, East Asia, and South America, due to its high seed oil content and significant economic value (Landoni et al., 2023). Castor seeds are considered representative of dicot endospermic seeds, with an embryo consisting of two thin, leaf-like cotyledons sandwiched between large endosperm tissues, all encased in a lignified and reinforced seed coat (Yu et al., 2019). Plant seed development begins with double fertilization and is influenced by communication between the embryo, endosperm, and maternal tissues (Bleckmann et al., 2014). Numerous transcription factors (TFs) from various families have been characterized as part of the complex regulatory network governing seed development, with MADS-box proteins emerging as key players.

*MADS-box* proteins are homeotic TFs involved in reproductive development across vascular plants, including flower development, inflorescence architecture, pollen development, and seed/fruit development (Dreni et al., 2007; Gramzow and Theissen, 2010; Smaczniak et al., 2012). These TFs are present and conserved across nearly all eukaryotic groups, from *Saccharomyces cerevisiae* to *Homo sapiens* (Shen et al., 2021). The acronym “MADS-box” is derived from the yeast MINI CHROMOSOME MAINTENANCE 1 (MCM1), the Arabidopsis AGAMOUS (AG), the Antirrhinum DEFICIENS (DEF), and the mammalian SERUM RESPONSE FACTOR (SRF) (Norman et al., 1988; Passmore et al., 1988; Schwarz-Sommer et al., 1990; Yanofsky et al., 1990). *MADS-box* genes are among the largest families of transcription factors in plants, with 107 members in *Arabidopsis*, 300 in Wheat, and 79 in peach (*Prunus persica*) (Riechmann et al., 2000; Parenicová et al., 2003; Wells et al., 2015; Schilling et al., 2020). These genes are characterized by a highly conserved *MADS-box* (M) domain in the N-terminal region, and could specifically bind to the CC(A/T)6GG sequence, known as the CArG-box motifs, found in the regulatory elements of their target genes (Hayes et al., 1988; Parenicová et al., 2003). Based on their C-terminal sequences, *MADS-box* genes are categorized into two distinct groups: type I and type II (Alvarez-Buylla et al., 2000). Type I genes are further subdivided into Mα, Mβ, and Mγ subgroups, whose function are not fully understood, although emerging studies suggest their involvement in regulating female gametophyte and endosperm development in *Arabidopsis* and grasses (Bemer et al., 2008; Colombo et al., 2008; Steffen et al., 2008; Roszak and Köhler, 2011; Shirzadi et al., 2011; Hehenberger et al., 2012; Chen et al., 2016; Batista et al., 2019; Paul et al., 2022; Zhang et al., 2023). Type II *MADS-box* genes, also referred to as MIKC-type genes, are distinguished by four typical protein domains: MADS (M), intervening (I), keratin-like (K), and C-terminal (C), and are further classified into MIKC* and MIKC_C_ subgroups (Kaufmann et al., 2005). *MADS-box* genes are central to the widely accepted ABCDE model of flower development, where they control floral organ identity and growth by forming multimeric complexes (Cheng et al., 2017). In *Arabidopsis*, class A MADS-box gene *APETALA1* (*AP1*); class B genes AP3 and *PISTILLATA* (*PI*); class C gene *AGAMOUS* (*AG*); class D genes *SEEDSTICK* (*STK*), *SHATTERPROOF1* (*SHP1*), and *SHP2*; and class E genes *SEPALLATA* (*SEP*) 1-4, are key regulators (Guo et al., 2015). Class A genes determine sepal identity, class A and B determine petal identity, class B and C together define stamen identity, class C alone specifies carpel identity. Class D genes are involved in ovule development, while class E genes function as “bridging molecules”, facilitating the specification of sepals, petals, stamens, and carpels by promoting protein-protein interactions (Immink et al., 2009). Several studies have shown that *MADS-box* genes are crucial for various physiological processes, including floral organ identity, ovules, and fruit development (Nam et al., 2003; Becker and Theißen, 2004; Ruelens et al., 2013). Recent researches have highlighted the role of *MADS-box* TFs in seed development and seed oil accumulation in Arabidopsis (Zhang et al., 2024b).

In this work, we identified 56 *MADS-box* genes in castor genome and performed phylogenetics, gene architecture, chromosomal distribution, evolutionary dynamics analyses to elucidate their evolutionary relationships at the genome level. Furthermore, tissue-specific expression and seed development profiles analysis revealed that *RcMADS16* and *RcMADS41* play essential roles in castor seed coat formation and endosperm development, respectively. Together, our analysis offers fresh perspectives on the possible role of these genes in castor seed development.

## Materials and methods

### Plant materials

Castor seeds from the inbred lines small-seed ZB306 and large-seed ZB107 were germinated in an incubator at 30°C for two weeks. Subsequently, the seedlings were subsequently moved to the greenhouse at Southwest Forestry University, where they were maintained under a 16-hour light/8-hour dark cycle. Seeds were collected at 5, 15, 25, 35, and 55 days after pollination (DAPs), and the seed coat were separated, frozen quickly in liquid nitrogen and kept at −80°C for storage.

### Identification and characterization of *MADS-box* genes in castor

Two *MADS-box* domains (PF00319 and PF01486) were used as queries to search the castor genome with HMMER (v3.3.2) (Wells et al., 2015). A local BLASTP search was then performed against all protein sequences of castor, using Arabidopsis *MADS-box* genes as queries with an E-value threshold of < 1e-10. The identified *MADS-box* genes were further analyzed using the Venny tool (https://bioinfogp.cnb.csic.es/tools/venny/). The ExPASy ProParam (https://web.expasy.org/) was employed to calculate the predicted isoelectric point (PI) and molecular weight (MW) of the candidate RcMADS proteins (Gasteiger et al., 2003). Subcellular localization was predicted by Plant-mPLoc v2.0 (http://www.csbio.sjtu.edu.cn/bioinf/plant-multi/).

### Phylogenetic analysis of *MADS-box* genes in castor

*MADS-box* protein sequences of Arabidopsis and castor were downloaded from the TAIR (https://www.arabidopsis.org/) and Euphorbiaceae Database (http://eupdb.liu-lab.com/), respectively. All these amino acid sequences were aligned with ClustalW2 (http://www.clustal.org/clustal2/). Phylogenetic trees were constructed using the maximum likelihood (ML) method with 1000 bootstrap replicates, and visualized using iTOL (https://itol.embl.de/) (Abdullah-Zawawi et al., 2021).

### Conserved domain and gene structure analyses

Conserved domains of individual *RcMADS-box* proteins were predicted using SMART program (http://smart.embl-heidelberg.de/). Gene structures were visualized using the Gene Structure Display Server (version 2.0) (http://gsds.cbi.pku.edu.cn/). Secondary structures were predicted using the NetSurfP-3.0 (https://dtu.biolib.com/NetSurfP-3/) (Lin et al., 2023), and the three-dimensional structures were predicted using AlphaFold3 (https://alphafoldserver.com/). The PDB files of these *MADS-box* proteins were visualized using PyMOL 3.0 software (https://pymol.org/#download), and the three-dimensional structures alignment also used this software (Jumper et al., 2021).

### Chromosomal distribution, gene synteny, and evolutionary analyses

Chromosomal locations of *RcMADS-box* genes were obtained from the castor genome and visualized using TBtools (Chen et al., 2023). Gene duplication events were detected using MCScanX (Wang et al., 2012). Tandem and segmental duplication gene pairs were extracted from the respective duplication files of the castor genome. Gene collinearity was visualized using jcvi (Wan et al., 2025). The *Ks* value of these collinearity gene pairs were calculated using the -ks, -kp programs of WGDI package (Sun et al., 2022).

### Expression profiling and gene co-expression analysis

RNA-seq raw data were generated from ten different tissues, including root, stem, leaf, pollen, inflorescence, capsule, seedling at three weeks post-planting, germinated seed, endosperm, ovule, and five stages of seed development (5, 15, 25, 35, and 55 DAPs, referred to as S1-S5). Quality control was conducted using fastp v0.24.0 (Chen et al., 2018). Clean reads were aligned to the newly published castor genome using HISAT2, and the gene expression levels were quantified with the FeatureCounts program in the Subread package (Kim et al., 2015; Liao et al., 2019). Tissues-specific expression profiles of all *RcMADS-box* genes were analyzed using the K-means clustering method and visualized with the pheatmap package in R (Xanthopoulou et al., 2022).

To investigate the role of *RcMADS-box* genes during castor seed development, genes co-expressed across the five seed developmental stages (from 15 to 55 DAPs) were identified using ClusterGVis (https://github.com/junjunlab/ClusterGVis). Subsequently, co-expression networks were constructed using the GENIE3 R package, based on the FPKM values of genes specifically expressed during the early and middle-late stages of seed development (Huynh-Thu et al., 2010). Gene interactions within these networks were analyzed and visualized using Cytoscape (v3.9.1) (Shannon et al., 2003). For the prediction of *cis*-acting elements in the promoter regions of genes involved in the networks, the 2000 bp upstream sequences from the translation start codons of each gene were extracted according to their genomic positions. Potential binding sites and their associated functions were identified using the New PLACE website (https://www.dna.affrc.go.jp/PLACE/?action=newplace), and the results were displayed using TBtools (Chen et al., 2023).

### Yeast one-hybrid assays

Yeast One-Hybrid (Y1H) assays were employed to determine the binding of *MADS-box* transcription factors (TFs) to the promoters of potential target genes. The promoter sequences were amplified from castor genomic DNA and construct the bait vectors, the promoters fragments of target genes *RcXYL1* (from -260 to -500 bp) and *RcPRX42* (from -700 to -1101 bp) containing the CArG cis-elements were cloned into the pAbAi plasmid. The full-length CDS sequences of hub *MADS-box* TFs, synthesized from the cDNA of castor seed total RNA, were recombined into the pGADT7 vector to generate the Prey-Effector plasmids. Y1H assays were carried out using the Yeast-One-Hybrid Media kit (Coolaber, China, YH1001-10T). Bait-Reportor plasmids were transformed into Y1H Gold yeast strains and selected on minimal synthetic dropout medium lacking uracil (SD/-Ura). Yeast cells grown in SD/-Ura liquid media were diluted to 10-, 100-, and 1000-fold concentrations. The optimal concentration of Aureobasidin A (AbA) for selection was determined by incubating bait strains in SD/-Ura solid medium with 0, 100, 300, 500, or 700 ng/mL AbA. The prey vectors were then transformed into all bait strains and grown on SD/-Ura/-Leu medium. The pGADT7 empty vector was used as negative control. All primers used for Y1H assays are listed in **Supplementary Table S1**.

### RNA isolation and quantitative real-time PCR

Total RNA from seed coats at different developmental stages was extracted using the RNAprep Pure Plant Plus kit (Tiangen Co. Ltd, Beijing, China; Catalog No. DP441), designed for samples rich in polysaccharides and polyphenolics. RNA quality and quantity were determined using a NanoDrop2000 Spectrophotometer (Thermo Scientific, USA). Complementary DNA (cDNA) was synthesized using the All-in-One First-Strand cDNA Synthesis SuperMixfor qPCR kit (TransGen Biotech, Beijing, China). Primers were designed using Primer3 (https://bioinfo.ut.ee/primer3/) with melting temperatures (Tm) ranging between 57°C and 59°C. qRT-PCR was performed on a CFX96 Touch Real-Time PCR Detection System (Bio-Rad, Berkeley, California), utilizing the PerfectStart Green qPCR SuperMix Kit (TransGen Biotech, Beijing, China). Each gene was tested in triplicate biological samples with three technical replicates per sample. Relative expression levels were calculated using the 2^-ΔΔCT^ method, with *RcActin* serving as the internal control. Primers sequences are provided in **Supplementary Table S2**.

## Results

### Identification of *MADS-box* genes in castor

A total of 57 *MADS-box* putative genes were identified in the castor genome via BLAST, while a further 26 genes containing both the MADS domain (PF00319) and K-box domain (PF01486) were detected through an HMMER search. An additional 30 genes containing only the MADS domain were also identified (**Supplementary Figure S1**). One gene that contained only the K-box domain was excluded, resulting in 56 *MADS-box* candidates, which were designated as *RcMADS1* to *RcMADS56* based on their chromosomal positions (**Supplementary Table S3**). The lengths of these genes varied from 470 to 35,338 bp, with an average length of 5,543.875 bp. Thirty-one genes were longer than 3,000 bp. The amino acid lengths ranged from 119 to 697 aa, with an average length of 280.86 aa; more than 80% of the proteins were between 200 and 400 aa (**Supplementary Table S3**). The theoretical molecular weight (Mw) of these proteins ranged from 14.11 to 80.00 kDa, and the isoelectric point (pI) ranged from 4.96 to 10.15 (**Supplementary Table S3**). Subcellular localization predictions indicated that approximately 89.29% (50 genes) of these *RcMADS* genes were located in the nucleus, while 5 genes were located to the chloroplasts, and one gene (*RcMADS37*) was predicted to reside in the cytoplasm (**Supplementary Table S3**).

### Phylogeny and classification of *RcMADS-box* genes

**Figure 1:**
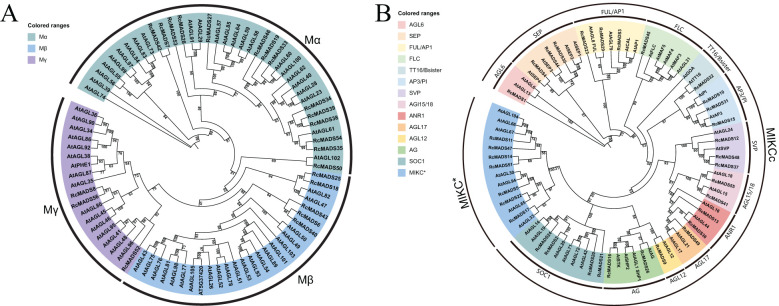
Phylogenetic analyses of *MADS-box* genes from castor and Arabidopsis. The Type I **(A)** *MADS-box* genes are divided into three subgroups, and Type II **(B)** are divided into two subgroups and 13 clades, each of these subgroups and clades are marked with different colors.

The identified *RcMADS-box* genes were classified into two major groups: type-I and type-II, containing 22 and 34 genes, based on phylogenetic analysis using Arabidopsis and castor *MADS-box* genes (**Figures 1A, B**). Type-I genes were further subdivided into the Mα, Mβ, and Mγ subgroups, containing 14, 5, and 3 genes, respectively. Phylogenetic analysis suggested that the divergence of type-I *MADS-box* genes between Arabidopsis and castor occurred prior to the separation of these two species (**Figure 1A**). Type-II genes were subdivided into the MIKCc and MIKC* subgroups, comprising 27 and 7 genes in castor, respectively (**Figure 1B**). The MIKCc subgroup could be further divided into 13 evolutionary clades based on the known Arabidopsis *MADS-box* genes, such as AGL6 (*RcMADS1*), SEP (*RcMADS4*/*30*/*44*), FUL/AP1 (*RcMADS3*/23/29), FLC (*RcMADS45*), TT16/Bsister (*RcMADS32*), AP3/PI (*RcMADS10/15/31*), SVP (*RcMADS12/37/48*), AGL15/18 (*RcMADS*49/55), ANR1 (*RcMADS13/36*), AGL17 (*RcMADS49*), AGL12 (*RcMADS9*), SOC1 (*RcMADS2/20/21/24*), and AG (*RcMADS16/26*). Genes from castor and Arabidopsis that clustered in the same clade likely share similar functions, providing important insights for the selection of candidate *RcMADS-box* genes in further studies on castor seed development.

### Conserved domains and structures of *RcMADS-box* genes

**Figure 2:**
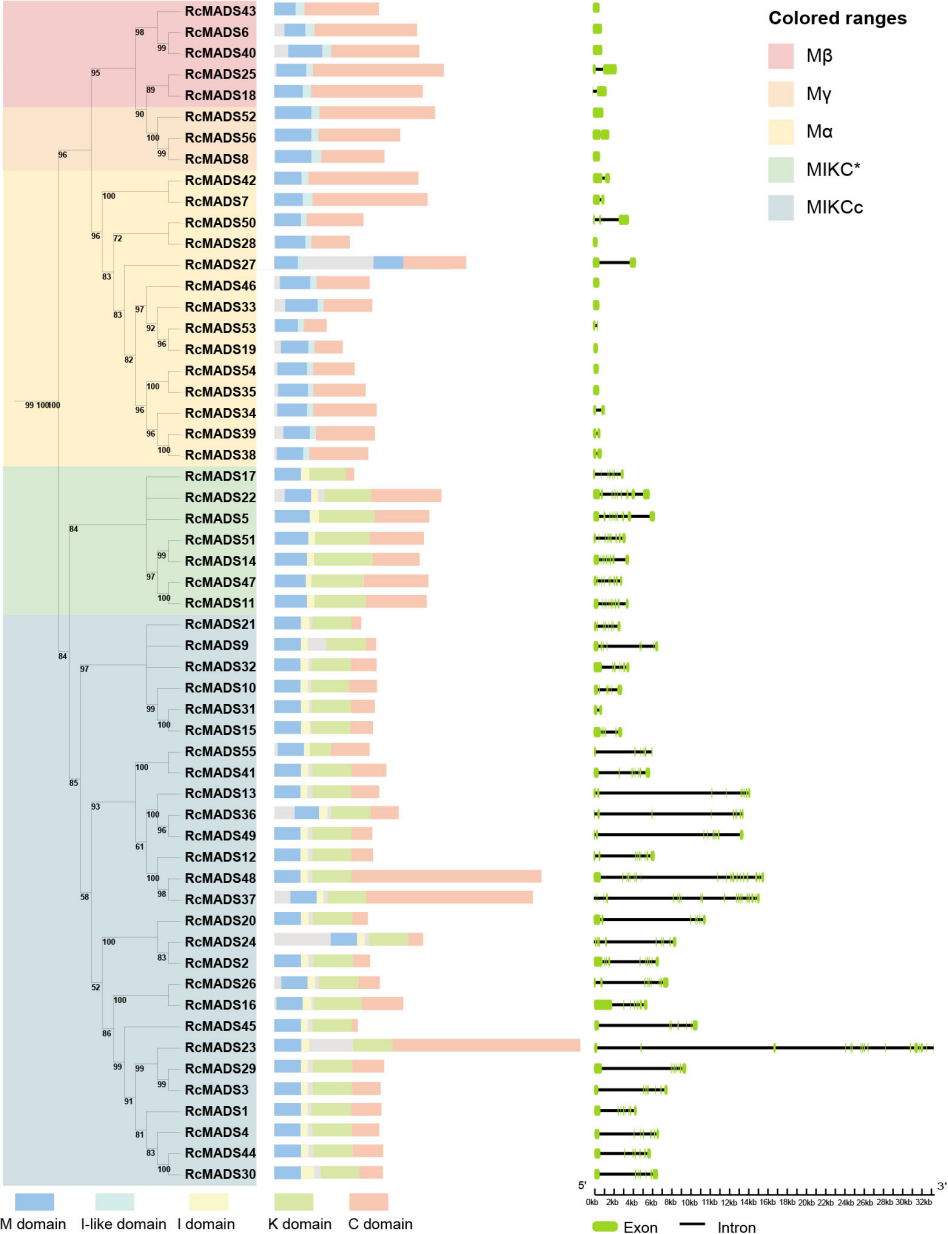
Phylogenetic tree, conserved domain, and gene structure of *MADS-box* genes in castor. The M, I-like, I, K, and C domain is represented as blue, cyan, yellow, green, and orange, respectively. The exon and intron is represented as green block and black line, respectively.

Conserved domain analysis revealed differences in the MADS (M) domain sequences between type-I and type-II RcMADS proteins in castor (**Figure 2**). Consistent with the above HMMER search results, the 22 type-I proteins contained only the M, Intervening-like (I-like), and C domains, whese the 34 type-II proteins included the M, I, Keratin-like (K), and C domains (**Figure 2**). Additionally, the intron-exon patterns of type-I genes were simpler than type-II genes. Notably, 12 out of the 22 type-I *RcMADS-box* genes were intronless, while none of the type-II lacked introns. In contrast, 26 of the 34 type-II *RcMADS-box* genes contained five to ten introns.

**Figure 3:**
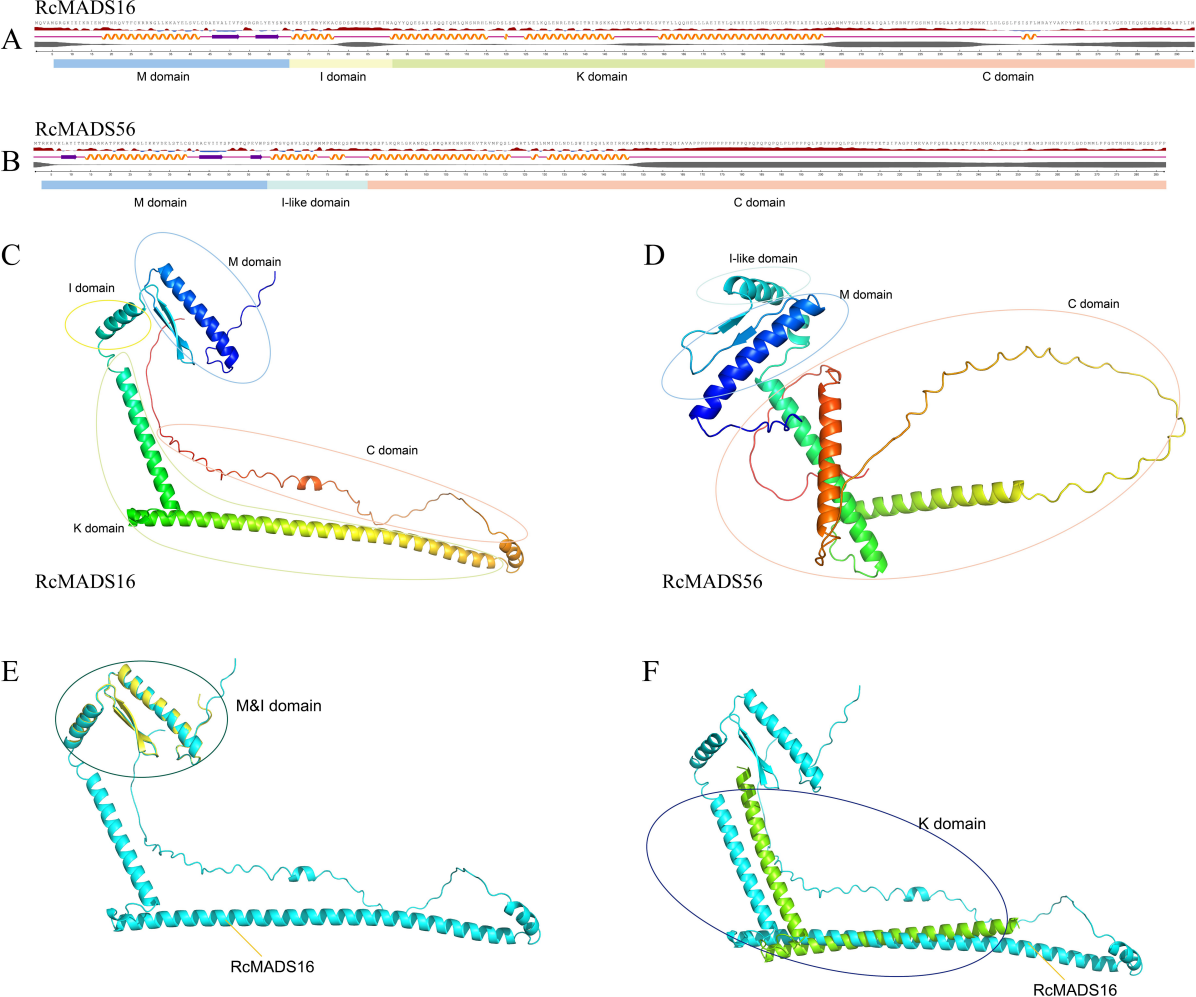
Protein structure prediction of representative *RcMADS* genes. *RcMADS16* and *RcMADS56* were selected to represent the type II and type I *MADS-box* genes, respectively. **(A, B)** shows the secondary structure and conserved domains of *RcMADS16* and *RcMADS56*. The orange helical line, purple arrow and purple line represents helices, strands and coils, respectively. **(C, D)** exhibits the tertiary structure of *RcMADS16* and *RcMADS56*. **(E)** Superimposition of the MADS (M) and intervening (I) domains shows the structural alignment between RcMADS16 (cyan) and its Arabidopsis homolog (yellow). **(F)** Structural alignment of the keratin-like (K) domain between RcMADS16 (cyan) and the Arabidopsis homolog (green)

The secondary and tertiary structures of RcMADS-box proteins were predicted using NetSurfP-3.0 tool and AlphaFold3, respectively (**Figure 3**). Each *RcMADS-box* gene exhibited similar secondary structures (**Supplementary Figures S2–S4**). The M domain, located at the N-terminal, consisted of an alpha helix (αA) and two antiparallel beta strands (βA and βB) (**Figure 3**). This region is essential for DNA binding, dimer and/or tetramer formation via the canonical CArG-box found in the upstream of all regulated genes. Several amino acid residues were highly conserved, including Arg (R), Lys (K), Glu (E), and Leu (L) at positions 24, 31, 34, and 38, respectively, in both type I and type II *RcMADS-box* proteins (**Supplementary Figure S5**). More conserved amino acid residues were detected in type II proteins (**Supplementary Figure S6**). The I/I-like domain, which contains an α-helix structure, determines DNA-binding specificity. This domain is weakly conserved, with characteristic residues such as Val (V) 64, Val (V) 67, Leu (L) 68, and Phe (F) 71 in the I domain of type I proteins, and Met (M) 63, Val (V) 66, Ile/Leu (I/L) 67, Arg (R) 69, Tyr (Y) 70 in the I-like domain of type II proteins (**Supplementary Figure S7**). The K domain, which mediates protein-protein interactions, consists of a series of helices. The C domain, composed of random coils, exhibits sequence variability (**Figure 3**; **Supplementary Figures S2–S4**). Homology modeling of the tertiary structures of *MADS-box* proteins from castor and Arabidopsis demonstrated high structural conservation within the same subfamily but considerable diversity between different subfamilies across different species (**Figure 3**).

### Chromosomal localization and gene duplications of *RcMADS-box* genes

**Figure 4:**
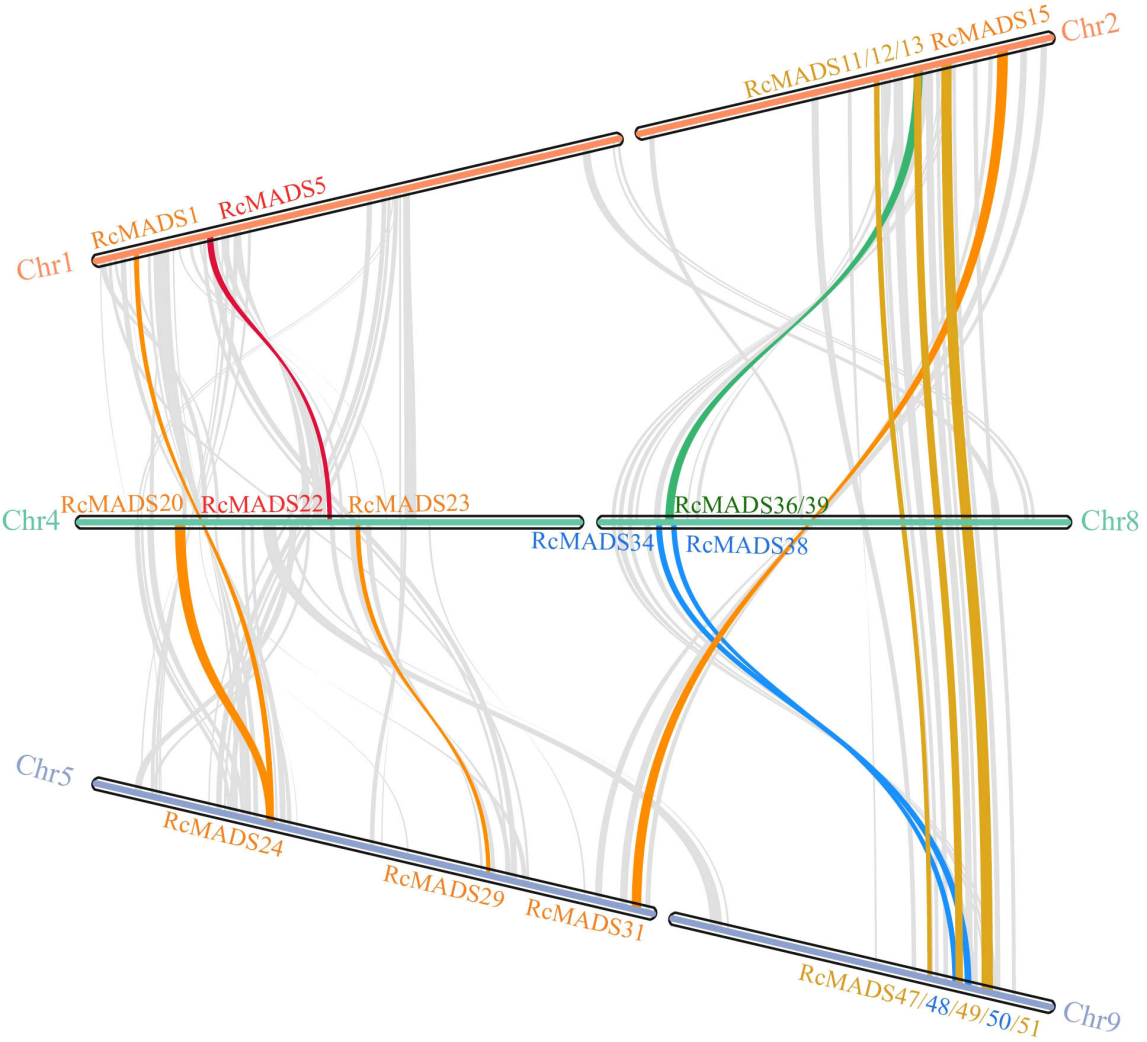
Gene duplication relationship of *MADS-box* genes in castor. Chromosomes are represented as horizontal bars, with gene names labeled at their respective positions. Gray curves in the background indicate syntenic relationships across the genome, while colored lines highlight different types of gene duplication events. These duplication patterns provide insights into the evolutionary expansion of the MADS-box gene family in castor.

The 56 *RcMADS-box* genes were unevenly distributed across nine of the ten chromosomes. Notably, there are nine *RcMADS-box* genes on Chr2, Chr8, and Chr9, while only two genes (*RcMADS32*/33) were located on Chr7. Gene duplication analysis using MCScanX revealed that five pairs of *RcMADS-box* genes arose through tandem repeats, including *RcMADS3*/*RcMADS4*, *RcMADS29*/*RcMADS30*, *RcMADS34*/*RcMADS35*, *RcMADS38*/*RcMADS39, RcMADS44*/*RcMADS45*, which were located on Chr1, Chr5, Chr8, and Chr9, respectively (**Supplementary Table S4**). Furthermore, a total of 14 gene pairs, including 23 *RcMADS-box* genes (including 2 type-I and 21 type-II *RcMADS-box* gene), representing 41.07% of the total *RcMADS-box* genes, were located in segmental duplicated regions (**Figure 4**; **Supplementary Table S4**). Notably, 10 out of 18 gene pairs belonged to the same subgroups or clades, suggesting that *RcMADS* genes were conserved during gene duplication. Evolutionary analysis revealed that the *Ka*/*Ks* ratios for all *RcMADS-box* gene pairs ranged from 0.09 to 0.32, all below 1, indicating strong purifying selection acting on these genes (**Supplementary Table S5**). Additionally, 9 out of 18 duplicated gene pairs had higher *Ks* values than the genome-wide average, suggesting duplication may accelerate gene evolution.

### Expression profiles of *RcMADS-box* genes in different tissues and stages of castor seed development

**Figure 5:**
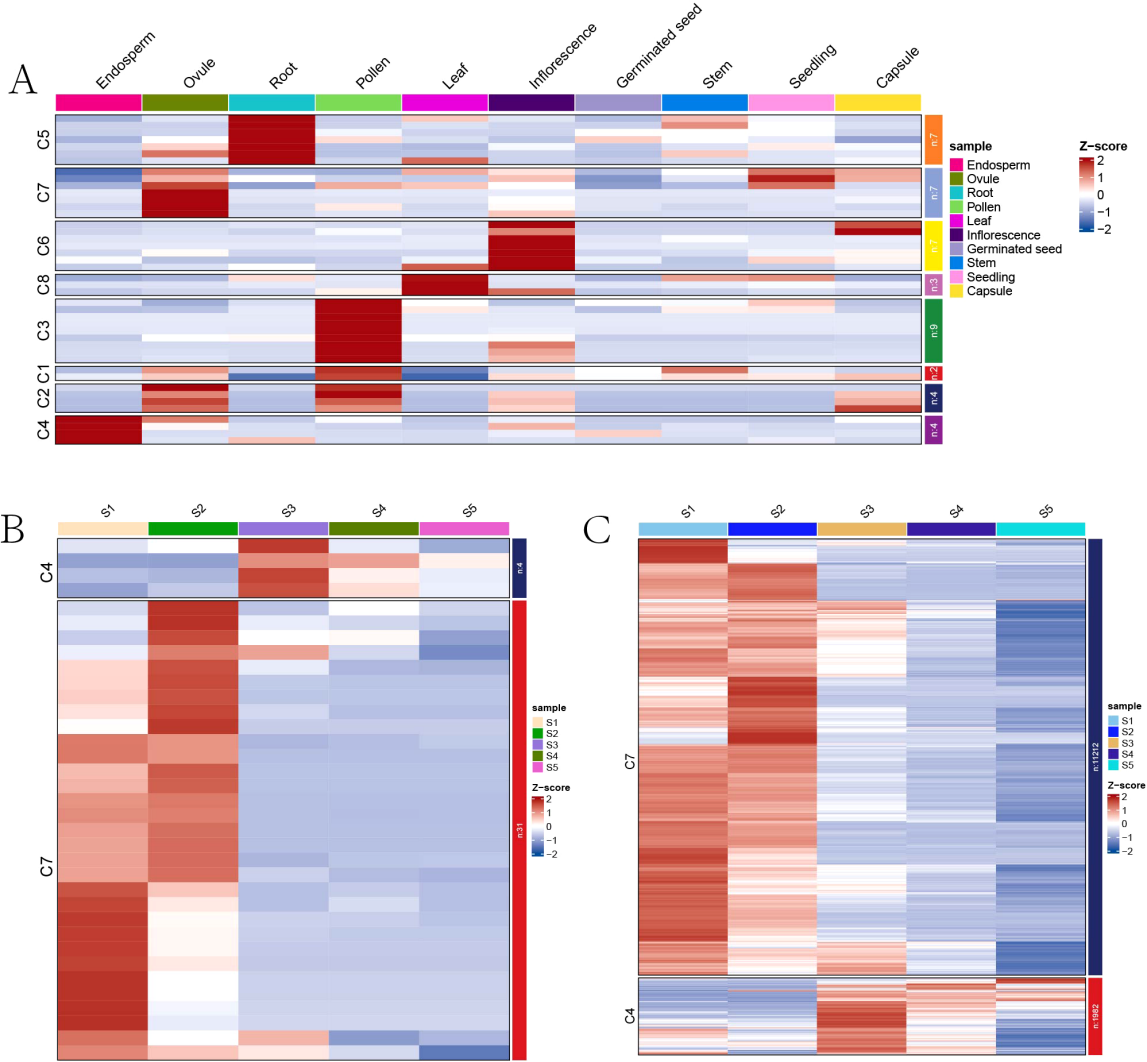
Expression profiles of *RcMADS-box* genes. **(A)** Expression patterns of *RcMADS-box* genes in different tissues of castor; **(B)** Expression patterns of *RcMADS-box* genes during five different development stages of castor seed; **(C)** Expression patterns of all seed-specific genes, including the *RcMADS-box* genes during the five seed developmental processes of castor.

To explore the expression profiles of *RcMADS-box* genes across various tissues, RNA-seq data were retrieved from the NCBI under the BioProject accessions PRJNA787114 and Euphorbiaceae database (http://eupdb.liu-lab.com/R_communis_browse/) (Liu et al., 2024), including tissues such as endosperm, ovule, root, pollen, leaf, inflorescence, germinated seed, stem, seedling, and capsule. Based on these expression profiles, a total of 14 *RcMADS* genes with FPKM values below 1 were excluded from the analysis, all of which were type-I, except for *RcMADS55* (**Supplementary Table S6**). The remaining 42 *RcMADS* genes were selected for k-means clustering analysis, and grouped into eight clusters (C1-C8) (**Figure 5A**). Two genes (*RcMADS22*/*38*) in C1 and four genes (*RcMADS26*/*30*/*44*/*56*) in C2 were highly expressed in both pollen and ovule tissues, while nine genes in C3 were predominantly expressed in pollen (**Figure 5A**; **Supplementary Table S6**). Seven genes in C5 were specially expressed in the root, and seven genes in C6 were highly expressed in inflorescence, while only three genes were specially expressed in the leaf. Notably, four genes (*RcMADS14*/*41*/*43*/*52*) in C4 and six genes (*RcMADS1*/*16*/*17*/*28*/*32*/*50*) in C7 were highly expressed in the endosperm and ovule, respectively.

We further analyzed gene expression across five stages of seed development (S1 to S5). Genes in C4 and C7 showed higher expression levels during the middle-late (S3-S5) and early-middle (S1-S3) stages during seed development, respectively (**Figure 5B**; **Supplementary Table S7**). Additionally, hierarchical clustering for seed-specific genes identified in our previous study (Yu et al., 2023) revealed that 11,212 genes were co-expressed with *RcMADS1*/*16*/*17*/*28*/*32*/*50*, which were clustered in C7, and highly expressed in the early stages of castor seed development (**Figure 5C**; **Supplementary Table S8**). A total of 1,982 genes co-expressed with *RcMADS14*/*41*/*43*/*52*, clustered in C4, were highly expressed in the middle-late stages of seed development (**Figure 5C**; **Supplementary Table S8**). Notably, four of the ten seed-specific genes belonged to type-I, while the remaining genes were type-II, suggesting that both types of *RcMADS* genes play essential roles during castor seed development.

### Prediction of gene regulatory networks during castor seed development

**Figure 6:**
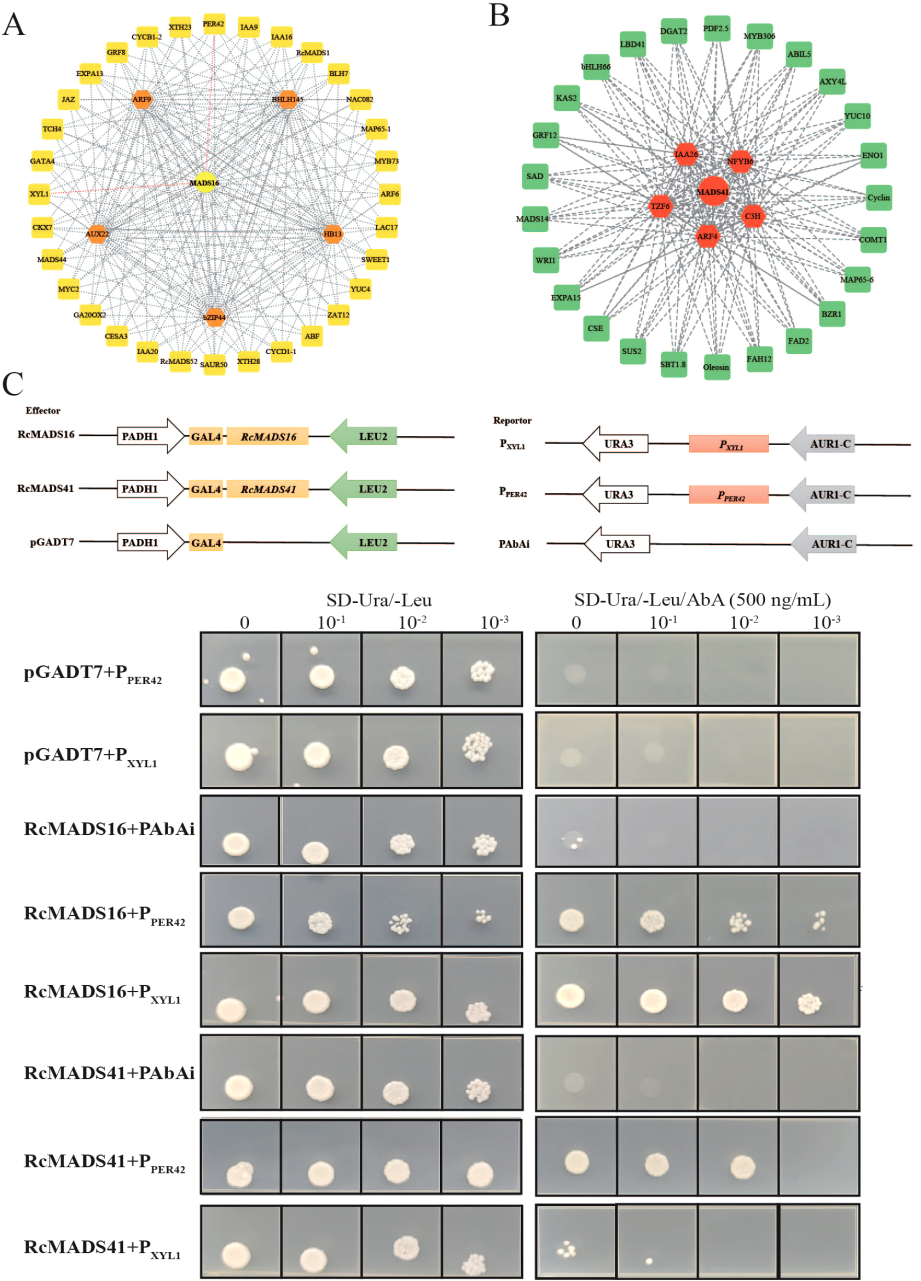
Prediction and verification of the *RcMADS16* and *RcMADS41* targeted genes during castor seed development. **(A)** Prediction of the *RcMADS16* regulatory network in the early seed developent stage. **(B)** Prediction of the *RcMADS41* regulatory network in the middle seed developent stage. **(C)** Y1H assays between *RcMADS16* and *RcMADS41* and the promoter fragments of the targeted genes.

We utilized GENIE3 to predict key regulatory factors and their downstream targets, and constructing two gene regulatory networks for early-middle and middle-late stages of castor seed development (**Figure 6A**). In the early stage, *RcMADS16* (the homolog of *AtSTK*) shared the maximum number of nodes with key TFs in the first layer, including *RcARF9* (*Rc05G010057*), *RcAUX22* (*Rc07G016821*), *RcHB13* (*Rc01G000650*), *RcbZIP44* (*Rc02G004777*), and *RcbHLH145* (*Rc02G004326*) (**Figure 6A**). GO enrichment analysis of *RcMADS16* target genes indicated that they were primarily involved in plant-type cell wall (GO: 0009505), xyloglucan 1,6-alpha-xylosidase activity (GO: 0080176), and regulation of RNA biosynthetic process (GO: 2001141) (**Supplementary Figure S8**). The promoter regions of these target genes contained numerous hormone response elements, including Cytokinin, GARE, SAUR, ERF3, ABA, Auxin/SA, as well as ARF elements (**Supplementary Figure S9**; **Supplementary Table S9**). Numerous CArG motifs, and TF binding elements (bZIP, MYB, HD, MYC, E2F, and SOC1) were also identified in their promoters (**Supplementary Figure S9**). These analyses suggest that *RcMADS16* regulates cell number and growth by modulating the expression of *RcCYCB1-2* (*Rc05G011661*), *RcCYCD1-1* (*Rc08G017624*), and *RcHB13* (*Rc01G000650*), and also influences secondary cell wall (SCW) biosynthesis through these lignin (*RcPER42*, *Rc05G009355*), cellulose (*RcCESA3*, *Rc08G018241*), and hemicellulose (*RcXYL1*, *Rc04G008432*) synthesis-related genes (**Figure 6A**). This finding suggests that *RcMADS16* and plant hormones, especially auxin, play a central role in regulating seed coat formation in castor.

During the middle-late stage, the *RcMADS41*-mediated regulatory network was explored. *RcIAA26* (*Rc07G016672*), *RcARF4* (*Rc01G001034*), *RcNFYB6* (*Rc10G023274*), *RcTZF6* (*Rc04G007632*), and *RcC3H* (*Rc03G005462*) were identified as the first-layer regulators (**Figure 6B**). KEGG enrichment of their target genes in the second layer revealed that they were predominantly involved in fatty acid biosynthesis (ko00061), plant hormone signal transduction (ko04075), and glycerolipid metabolism (ko00561) (**Supplementary Figure S10**). The *cis*-elements of all genes in this network were similar to those described above, except for the 2S (short for 2SSEEDPROTBANAPA) *cis*-element (**Supplementary Figure S11**, **Supplementary Table S10**), which is conserved in many storage protein gene promoters. Notably, the CArG motif was detected in nearly all target genes (28 out of 31), including *RcWRI1* (*Rc08G018567*), *RcDGAT2* (*Rc05G012439*), and *RcFAD2* (*Rc06G014656*), which are key TFs/genes involved in fatty acid accumulation.

To verify the potential interactions between these hub TFs and their target genes, the yeast one hybridization (Y1H) system was employed to test whether *RcMADS16* and *RcMADS41* could directly bind to the promoter of *RcPER42* and *RcXYL1*, two genes related to cell wall formation (**Figure 6C**). Compared with the negative control, yeast strains transformed with *RcMADS16* in pGADT7 and *P_XYL1_* in pAbAi vectors grew normally on the screening medium. However, when the prey was *P_PER42_*, yeast growth was inhibited, suggesting that *RcMADS16* directly binds to the *RcXYL1* promoter, but with weaker binding strength to the *RcPER42* promoter (**Figure 6C**). Conversely, when *RcMADS41-*pGADT7 and *P_XYL1_*-pAbAi or *P_PER42_*-pAbAi were transformed into yeast, the strains showed poor growth on medium supplemented with 500 ng/mL AbA, indicating that *RcMADS41* did not activate these promoters. These results suggest that *RcMADS16* is a key TF governing seed development by regulating the lignification of the seed coat, while *RcMADS41* likely plays an essential role in oil accumulation during castor seed development.

### qRT-PCR validation

**Figure 7:**
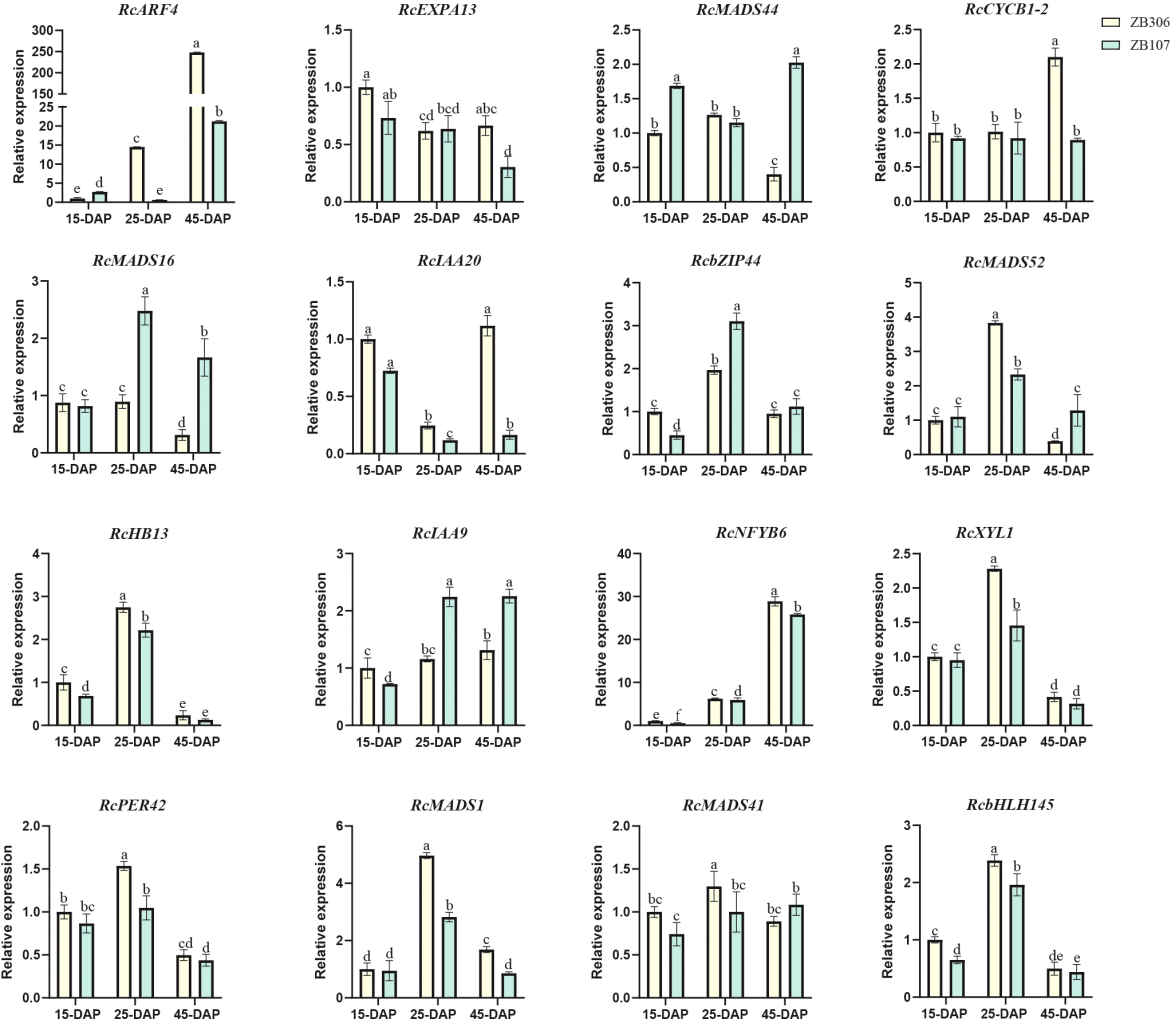
Relative expression levels of *RcMADS-box* and their targeted genes during castor seed coat development. The expression of each gene in ZB306 at 15 DAP was set as 1.0. The different letters represent statistical difference based on LSD (least significant difference) analysis with p<0.05. The relative expression levels are means ± SD (n=3).

To verify the reliability of RNA-Seq data and examine the differential expression levels between large-seed ZB107 and small-seed ZB306 during seed coat formation (**Figure 7**), 16 candidate genes were selected for qRT-PCR analysis. Compared to large-seed ZB107, ten of these genes exhibited higher expression levels in the seed coat of small-seed ZB306 during seed development, excluding *RcMADS16*/*44*, *RcbZIP44*, and *RcIAA9* (**Figure 7**). Notably, *RcXYL1* and *RcPER42* showed higher expression levels at the middle stage (25 DAP) of seed coat development, with their relative expression levels being higher in ZB306 than in ZB107. This supports previous findings that the lignin content in the seed coat ZB306 is higher than in ZB107 (Yu et al., 2019). Additionally, we confirmed the co-expression patterns between *RcMADS16* and *RcXYL1*, as well as *RcPER42* (**Figure 7**). Furthermore, the relative expression levels of selected genes verified by qRT-PCR were consistent with the mRNA abundance profiles from the RNA-Seq analysis.

## Discussion

The model dicotyledonous oil crop, castor, has evolved a unique seed morphology, characterized by a hard seed coat and persistent endosperm (Anjani, 2012). Although secondary cell wall components and important phytochemicals such as flavonoids and lignin have been identified in castor seed coats, little is known about the molecular mechanisms underlying seed coat identity (Tobimatsu et al., 2013; Wang et al., 2020a).

The *MADS-box* gene family is one of the most ancient and extensively studied transcription factor families across various species. Their functions were diverse, including roles in flowers formation, flowering time control, organ identity, and plant development (Theißen et al., 2016). However, the roles of *MADS-box* proteins in seed development have mainly been explored in Arabidopsis and rice. It’s well established that type-I *MADS-box* genes play essential roles in endosperm formation and development in Arabidopsis through imprinted or non-imprinted patterns (Zhang et al., 2018; Wang et al., 2020b; Dziasek et al., 2024). Several endosperm-related type-I *MADS-box* genes have been identified, i.e., *AGL9*, *AGL61*, *AGL62*, *AGL80*, and *PHE1* (Kang et al., 2008; Steffen et al., 2008; Zhang et al., 2024a). In our analysis, type-I genes *RcMADS28*/*43*/*50*/*52* were also highly expressed during the early stage of seed development when endosperm proliferation and cellularization predominantly occur (**Figure 5**), suggesting their potential functions in castor seed development.

In this study, we conducted a systematic analysis of the *MADS-box* family genes in the castor genome. A total of 56 *MADS-box* genes were identified, a lower number than the 107 and 75 genes found in Arabidopsis and rice, respectively (Parenicová et al., 2003; Arora et al., 2007). Notably, 33 out of the 56 *RcMADS-box* genes expanded through tandem and segmental duplication (**Figure 4**). The relatively low number of genes in castor were can be attributed to the absence of a species-specific whole-genome duplication event in castor, compared to Arabidopsis and rice (Xu et al., 2021). The structures of RcMADS-box proteins were characterized by the M, I, K, and C domains. The M domain, in particular, was highly conserved among all *MADS-box* genes in castor and is critical for the proper functioning of MADS-box TFs by enabling them to bind specific genes or gene regions (**Figure 2**). Consistent with previous studies in other species, these *MADS-box* genes in castor could be categorized into two main types: 22 type I (Mα, Mβ, Mγ) and 34 type II (MIKC_C_, MIKC*) genes. Among these, the 27 MIKC_C_ proteins were further divided into 13 clades based on their phylogenetic relationships (**Figure 1A**). Genes in the AG and SEP clades are the most extensively studied, corresponding to the D and E function genes in the well known ABC(DE) models of flower development (Dreni and Ferrándiz, 2022). For example, genes in the D class, including *STK*, *SHP1*, and *SHP2*, have been implicated in fruit and seed development (Dreni and Kater, 2014; Fei et al., 2021; Paolo et al., 2021). In Arabidopsis, *STK* has been identified as a master regulator of ovule identity, and *AGL11* in grape was essential for seedless fruit morphogenesis (Ezquer et al., 2016). The variation of the *STK* homolog in the oil palm, *SHELL*, is associated with the presence and thickness of the lignified shell (Malabarba et al., 2017). In Arabidopsis, *STK* regulates seed size by modifying the expression of the cell wall degradation gene *α-XYLOSIDASE1* (*XYL1*) (Di Marzo et al., 2022). It also controls fruit size by regulating cytokinin levels and the expression of *FRUITFULL* (*FUL*) in Arabidopsis (Marzo et al., 2020). In our study, *RcMADS16*, a homolog of *STK*, was highly expressed in the early stage of castor seed development, particularly in the seed coat, suggesting that *RcMADS16* is a master regulator of seed coat formation in castor, controlling processes such as cell cycle progression, plant hormone signal transduction, and secondary cell biosynthesis (**Figure 6**). Furthermore, *RcMADS16* exhibited significantly higher expression levels in the large-seed genotype (ZB107) compared to the small-seed genotype (ZB306) (**Figure 7**). This expression pattern suggests that *RcMADS16* plays a pivotal role in determining seed size, likely by regulating seed coat development. Given that our Y1H assays demonstrated direct binding of *RcMADS16* to the promoters of *RcXYL1* and *RcPER4*2—two genes potentially involved in cell wall remodeling and lignification—*RcMADS16* may influence seed coat thickness and composition (**Figure 6**). However, the precise molecular mechanism remains to be elucidated. Future studies could incorporate functional validation approaches, such as overexpression or knockout of *RcMADS16* in castor or heterologous systems, to directly assess its impact on seed coat formation, lignin deposition, and possibly oil accumulation. Additionally, further investigation into potential allelic variation in *RcMADS16* between the two genotypes could provide deeper insights into its role in seed size determination at the genetic level.

## Conclusions

In this study, we identified a total of 56 *MADS-box* genes in the castor genome, which can be classified into two main groups, each featuring conserved domains. Gene duplication analysis revealed that segmental duplication was the primary driving force behind the expansion of *RcMADS-box* genes, and the *Ka*/*Ks* ratio suggested that these genes have undergone purifying selection. Expression profiles showed that *RcMADS-box* genes were widely expressed in different tissues of castor, with 10 genes specifically expressed in castor seeds. Based on the results from co-expression networks, promoter analysis, Y1H, and qRT-PCR, *RcMADS16* and *RcMADS41* were identified as key regulators of castor seed development, with *RcMADS16* associated with seed coat formation and *RcMADS41* linked to oil accumulation. In summary, this study contributes to the understanding of the roles of MADS-box family genes in castor and provides a solid foundation for further investigations into their functions in seed or other organs development.

## Data Availability

The original contributions presented in the study are included in the article/**Supplementary Material**. Further inquiries can be directed to the corresponding authors.
