# Supplementary material for: Characterization analyses of MADS-box genes highlighting their functions with seed development in Ricinus communis
Source: Front Plant Sci. 2025 May 14;16:1589915. doi: 10.3389/fpls.2025.1589915 (PMC12116605; doi:10.3389/fpls.2025.1589915)
Supplement: Supplementary file 2 [file SupplementaryFile1.docx]

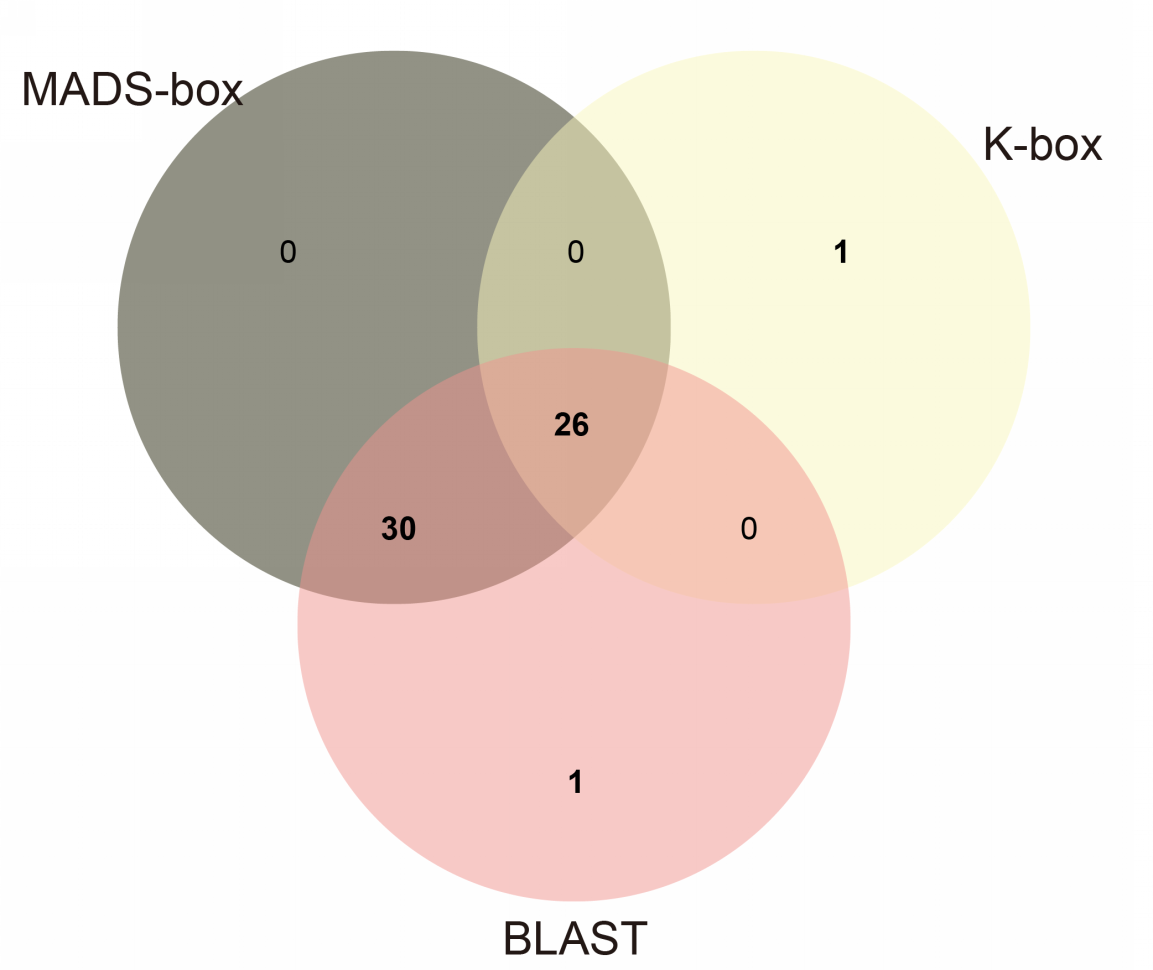


**Figure S1.** The overlapped genes of HMMER search and BLASTP reasults.


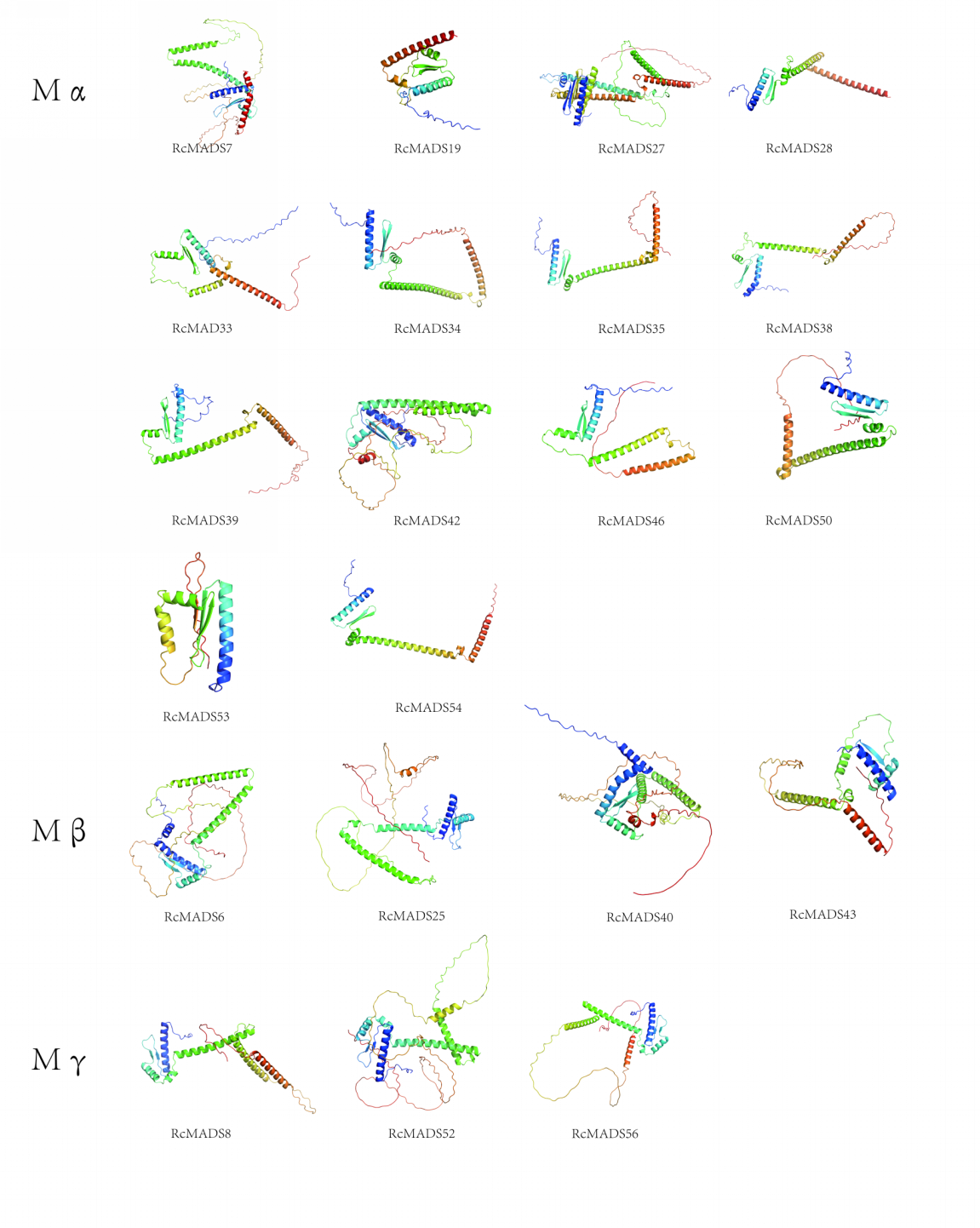


**Figure S2.** Prediction of tertiary structures of type I MADS-box genes in castor bean.


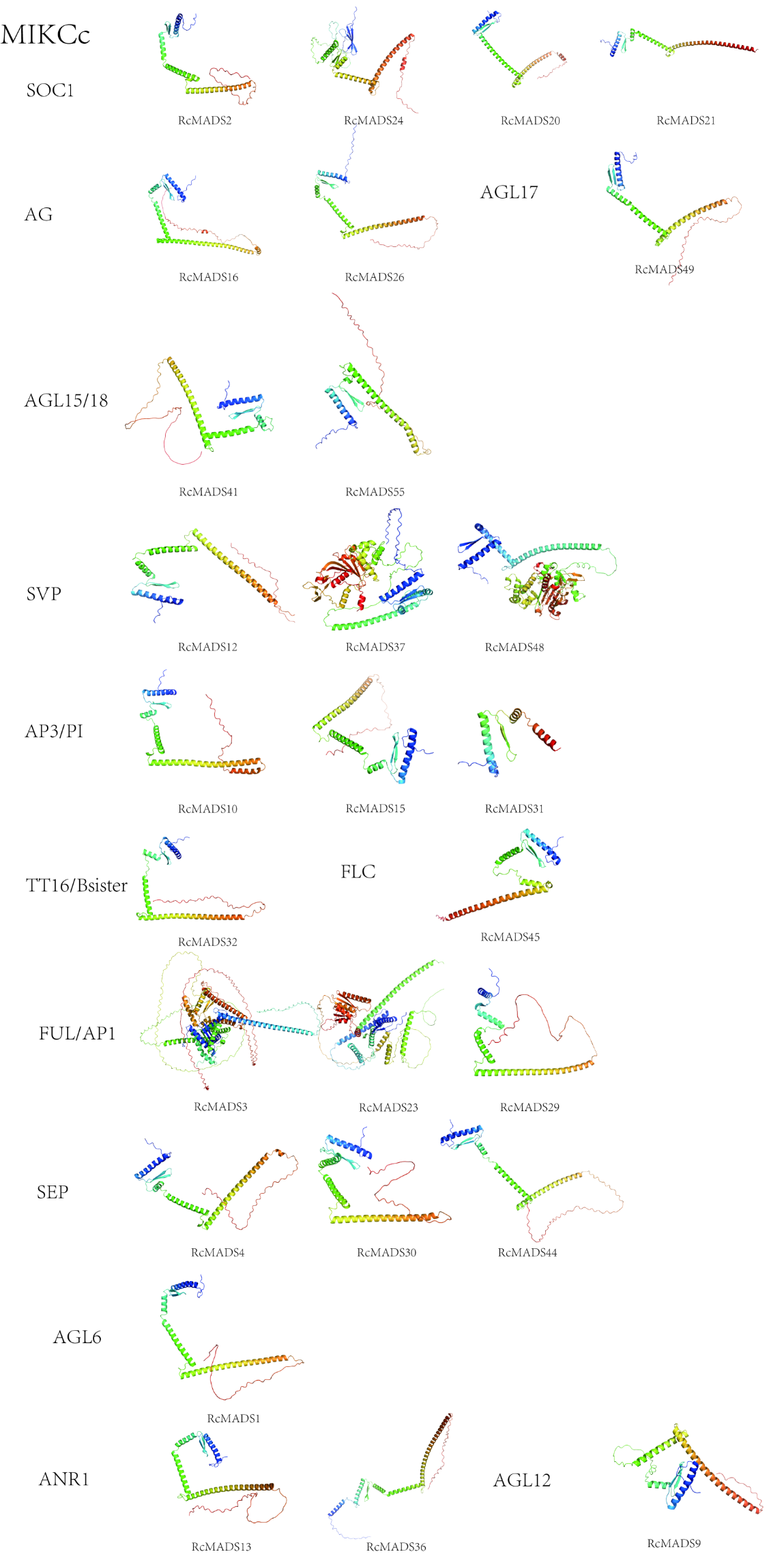


**Figure S3.** Prediction of tertiary structures of type II-MIKCc MADS-box genes in castor bean.

**
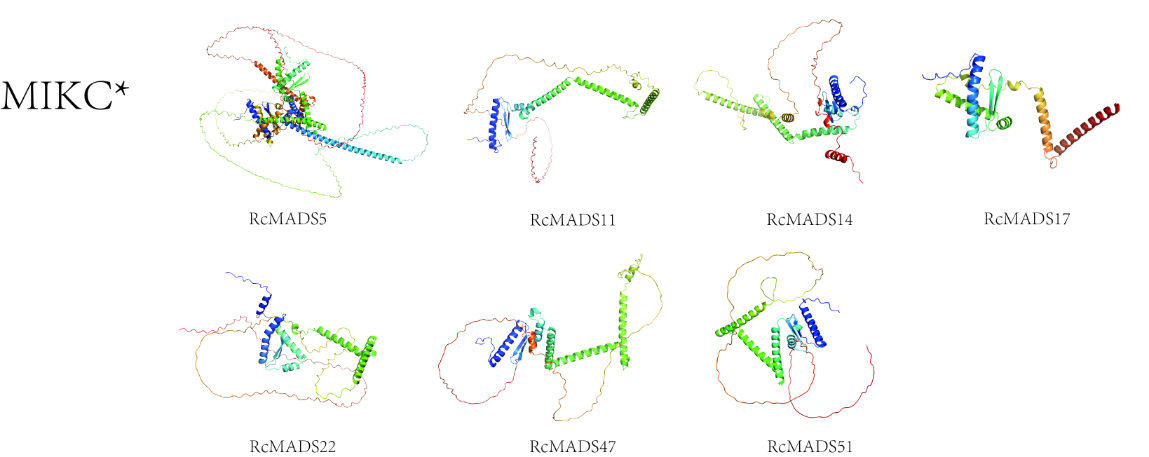
**

**Figure S4.** Prediction of tertiary structures of type II-MIKC***** MADS-box genes in castor bean.

**Figure S5.** Alignment the M-domain sequences of all castor genes. The blue box noted the conserved DNA binding domain, and the upper helix showed the secondary structure of RcMADS16.
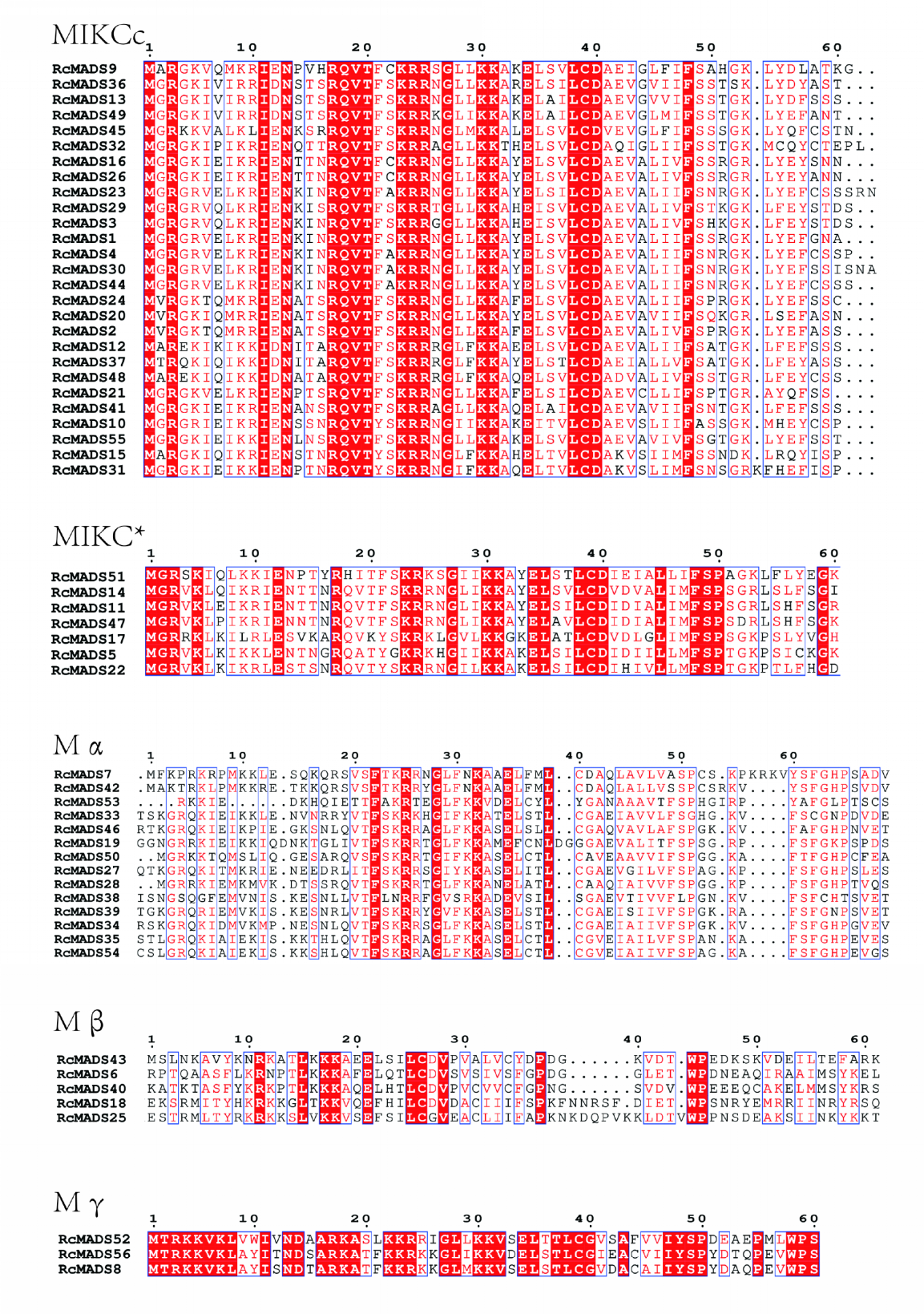


**Figure S6.** Multiple sequence alignment of M-domain of all castor MADS-box genes.


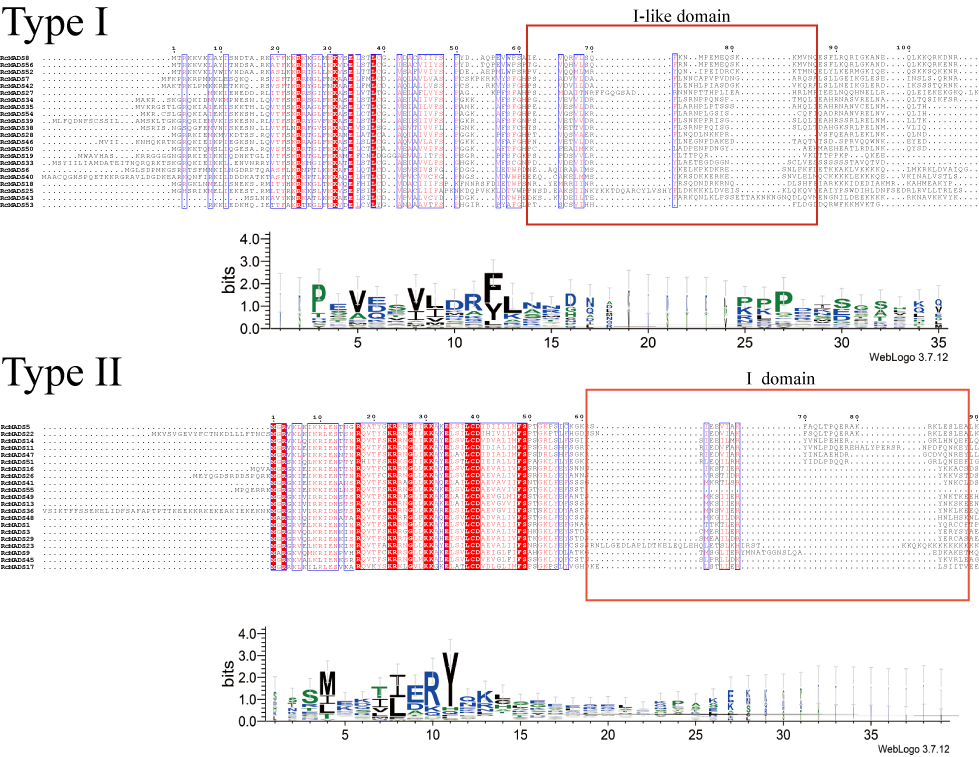


**Figure S7.** Alignment the I-domain sequences of type I and type II castor genes.

**Figure S8.** GO enrichment ananlysis of *RcMADS16* target genes


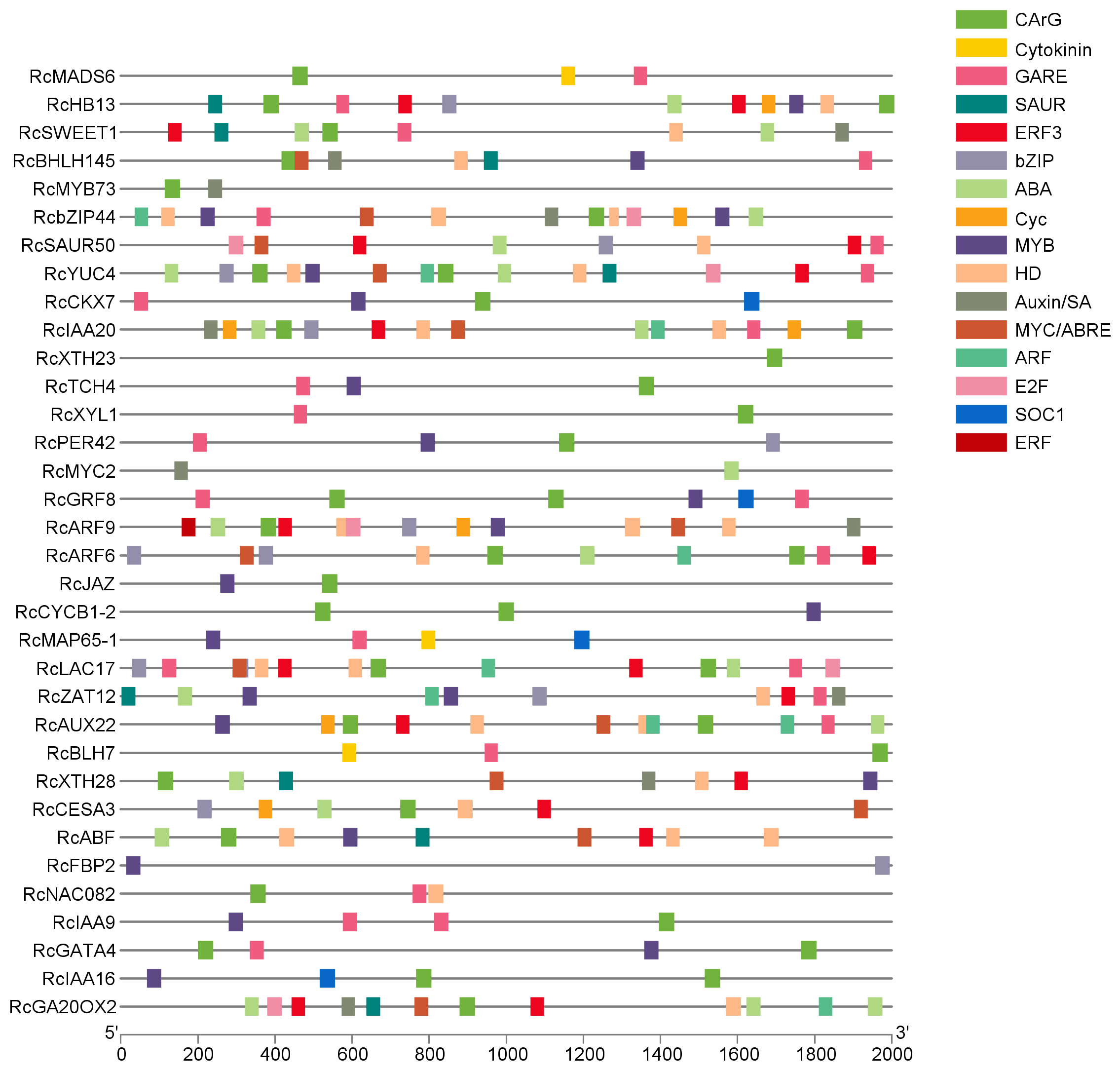


**Figure S9****.** Cis-elements in the promoters of MADS16 target genes


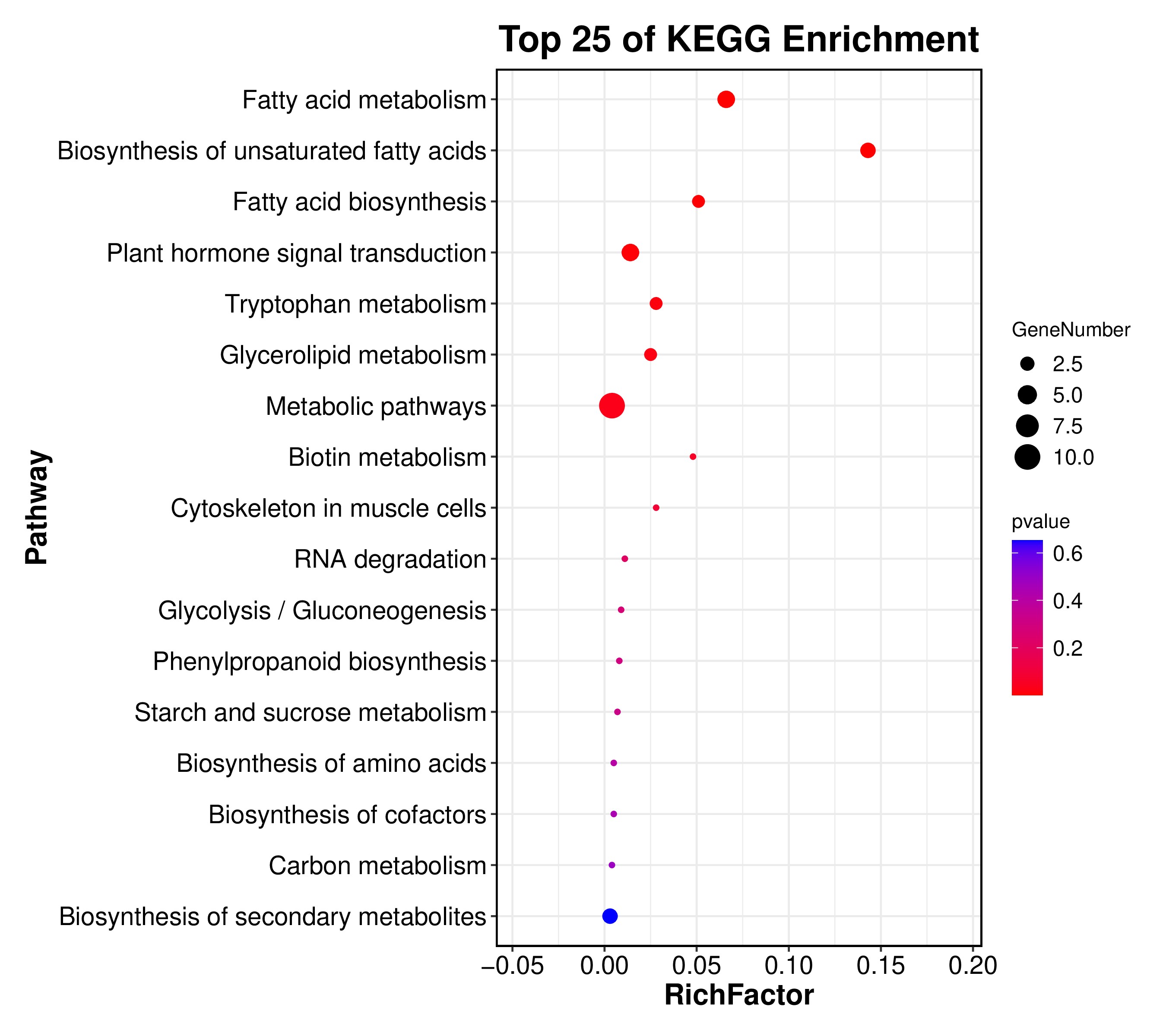


**Figure S10.** KEGG enrichment ananlysis of *RcMADS41* target genes

**
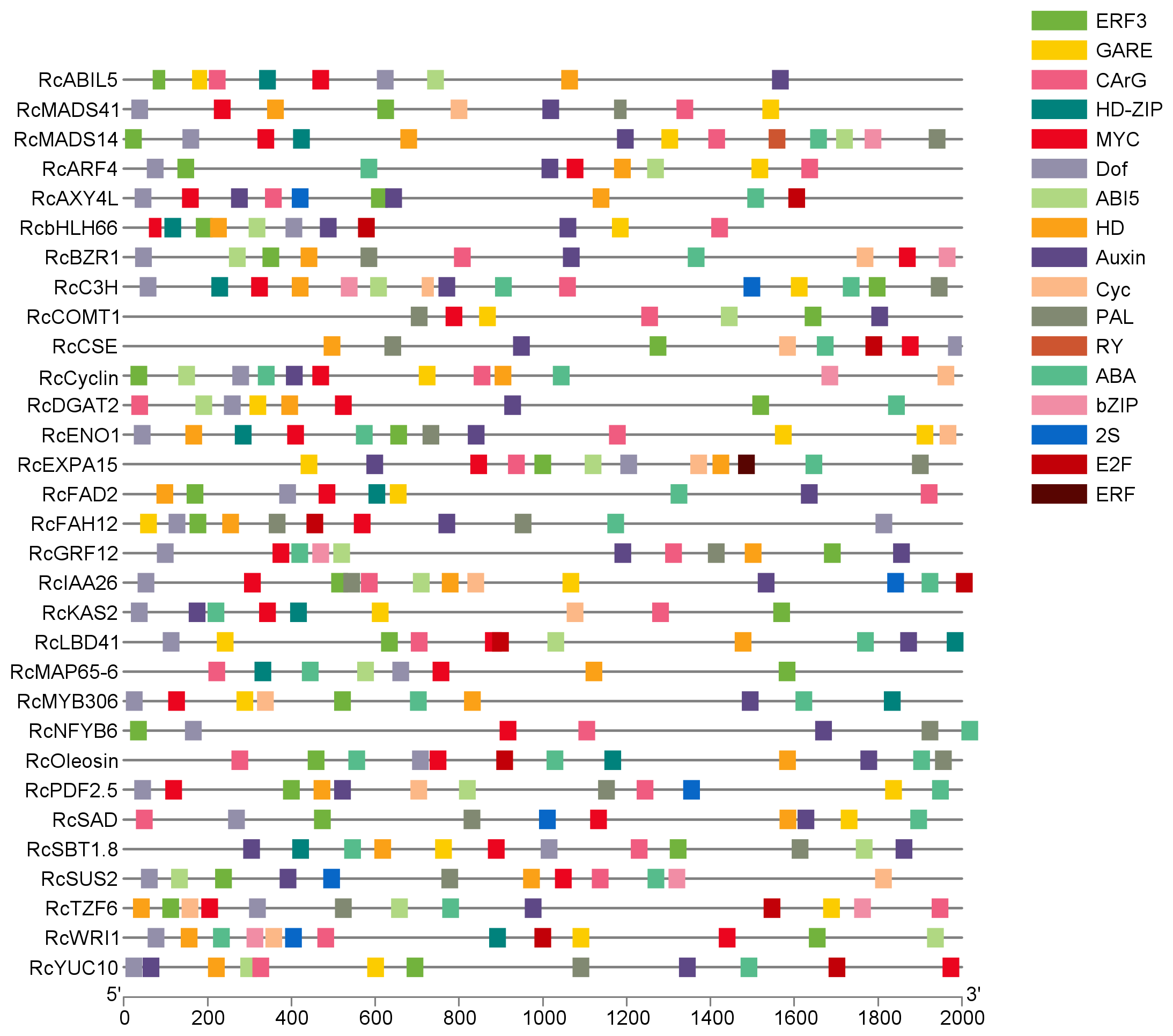
**

**Figure S11.** Cis-elements in the promoters of MADS41 target genes
